# Prospective Approach to the Anaerobic Bioconversion of Benzo- and Dibenzothiophene Sulfones to Sulfide

**DOI:** 10.3390/molecules24091736

**Published:** 2019-05-04

**Authors:** Olga Senko, Olga Maslova, Marina Gladchenko, Sergey Gaydamaka, Argam Akopyan, Sergey Lysenko, Eduard Karakhanov, Elena Efremenko

**Affiliations:** 1. Faculty of Chemistry, Lomonosov Moscow State University, Moscow 119991, Russia; senkoov@gmail.com (O.S.); gladmarina@yandex.ru (M.G.); s.gaidamaka@gmail.com (S.G.); arvchem@yandex.ru (A.A.); ls@oil.chem.msu.ru (S.L.); kar@petrol.chem.msu.ru (E.K.); 2 Emanuel Institute of Biochemical Physics, Russian Academy of Science, Moscow 119334, Russia

**Keywords:** organic sulfones biodegradation, desulfurization, anaerobic biotechnology, methanogenesis, immobilized cells

## Abstract

Sulfur recovery from organic molecules such as toxic sulfones is an actual problem, and its solution through the use of environmentally friendly and nature-like processes looks attractive for research and application. For the first time, the possible bioconversion of organic sulfones (benzo-and dibenzothiophene sulfones) to inorganic sulfide under anaerobic conditions with simultaneous biogas production from glucose within a methanogenesis process is demonstrated. Biogas with a methane content of 50.7%–82.1% was obtained without H_2_S impurities. Methanogenesis with 99.7%–100% efficiency and 97.8%–100% conversion of benzo- and dibenzothiophene sulfones (up to 0.45 mM) to inorganic sulfide were obtained in eight days by using a combination of various anaerobic biocatalysts immobilized in a poly(vinyl alcohol) cryogel. Pure cell cultures of sulfate-reducing bacteria and/or H_2_-producing bacteria were tested as additives to the methanogenic activated sludge. The immobilized activated sludge “enhanced” by bacterial additives appeared to retain its properties and be usable multiple times for the conversion of sulfones under batch conditions.

## 1. Introduction

A number of industrial and environmental problems associated with the possible presence of sulfur compounds in fuel are still not completely resolved. In this regard, the development of new, efficient, environmentally friendly, and cost-effective methods for the desulfurization of hydrocarbon raw materials remains relevant [1,2]. The problem associated with the control and management of chemical transformations of S-containing molecules is important, because the sulfur compounds present in fuel, even in low concentrations, lead to the inactivation of chemical catalysts and rapid equipment wear. In the composition of organic molecules, sulfur is most often found in the form of mercaptans, sulfide, disulfides, thioethers, sulfoxides, sulfones, thiophene, sulfoacids, etc. [3]. Biodesulfurization is a promising alternative to the traditional chemical method of controlling chemical transformations of S-containing molecules; it is based on the use of living aerobic (oxidative pathway: 4S and Kodama) or anaerobic (reductive pathway: C–S Cleavage) bacterial cells [1,2,4].

Oxidative 4S-pathway is the most widespread today. In it, sulfate is the final S-containing compound obtained after four chemical steps of transformation. The disadvantage of this process is that during desulfurization, a large consumption of oxygen is required, and a significant amount of carbon dioxide is released. In addition, the source and intermediate products significantly inhibit the activity of biocatalysts [5,6].

Toxic organic sulfones and sulfoxides, as is known, can be formed in the processes of conversion of petroleum feedstock, for example, during the leakage of petroleum products [7] and during the combustion of coal [3]. Thermal decomposition of the sulfone-containing compounds that is used to remove sulfur in the form of SO_2_ could be a part of the final recovery step in an oxidative desulfurization process [8], but the thermal stability of sulfones depends upon the chemical environment of the sulfone group. In contrast to aliphatic sulfones, the thermal decomposition of sulfones flanked by aromatic groups, such as thianaphthene 1.1-dioxide and dibenzothiophene 5.5-dioxide, is accompanied by either partial elimination of the sulfone group or formation of S-containing polycyclic compounds. So, for decomposition of benzo- and dibenzothiophene sulfones, the approach based on the use of biocatalysts under anaerobic conditions may be more effective.

Sulfate-reducing bacteria (SRB), such as *Desulfovibrio sp*., can be used as the main biocatalyst, providing the removal of sulfur from organic compounds in the form of sulfide under anaerobic conditions. Today, Green Chemistry considers sulfide-containing wastes and renewable monomers as promising raw materials for the production of new polymers and functional materials [9]. Currently, the process to obtain biogas as a commercial product with the biological treatment of biodegradable wastes under anaerobic conditions is already well developed [10]. For these purpose, various variants of natural anaerobic sludge (AS) are most often used. All samples of AS usually are bacterial consortia and contain, in addition to archaea-producing methane, a number of other cells, including SRB and H_2_-producing bacteria (for example, *Clostridium sp*.) [11].

Today, it is known that methanogenesis and retrieving of sulfur from sulfate can be carried out simultaneously using the same anaerobic sludge [12]. Such processes are attractive, both from an environmental and from an economic point of view, since they make it possible to obtain commercial products (biogas and sulfides) during waste conversion within a single processing.

**Figure 1:**
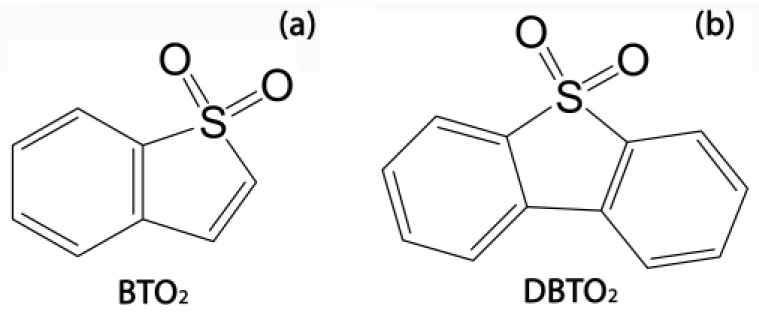
Chemical structures of organic sulfones used in the study: benzothiophene sulfone (BTO_2_) (**a**) and dibenzothiophene sulfone (DBTO_2_) (**b**).

To obtain biogas without H_2_S impurities, it is advisable to carry out the processes of conversion of organic sulfur-containing compounds at a pH not lower than 7.2, since sulfides will remain in dissolved form or as precipitates at such pH. Such conditions can be provided by using a buffer or by introducing additional cells as biocatalysts from the outside into the natural anaerobic consortia. Thus, it is possible to shift the biochemical conversion pathways towards the formation of hydrogen and sulfides, while decreasing the overall productivity of acidogenic bacteria that are part of the methanogenic consortia and produce acids lowering the pH of the media. This was taken into account in this work aimed at studying the possibilities of anaerobic conversion of S-containing organic compounds under anaerobic conditions in the frame of a methanogenic process using two sulfones: benzothiophene sulfone (thianaphthene 1.1-dioxide) (BTO_2_) and dibenzothiophene sulfone (dibenzothiophene 5.5-dioxide, DBTO_2_) (Figure 1).

Since S-containing organic compounds are toxic to cells applied as biocatalysts in bioconversion processes, it is advisable to use microorganisms in specially stabilized forms. Microbial cells immobilized in polyvinyl alcohol cryogel (PVA) are successfully used in the biocatalytic transformation processes of a wide range of wastes [13]. It is known that the use of natural anaerobic consortia (sludge) immobilized in a PVA cryogel allows to intensify the processes of methanogenesis, also in the presence of 10 ppm methiocarb sulfone [14].

Thus, the purpose of this work was to estimate the prospects of simultaneous anaerobic bioconversion of BTO_2_ and DBTO_2_ to sulfide with the production of biogas using different natural anaerobic methanogenic consortia with and without the addition of other anaerobic cells to the reaction medium. In order to increase the stability of cells to the toxic effect of organic sulfones, the consortia and cells of individual cultures were immobilized into a PVA cryogel. For a comparative analysis, both immobilized and suspension forms of AS were tested in the experiments.

## 2. Results

### 2.1. Biotransformation Capabilities of Organic Sulfones to Sulfide under the Action of Anaerobic Biocatalysts

It was shown (Table 1) that using AS as a biocatalyst for eight days it was possible to obtain an almost complete conversion of both sulfones (BTO_2_ and DBTO_2_) at their initially introduced concentrations of 0.15 and 0.45 mM (25–97 ppm). However, sulfide yield after 18 days, did not always reach the value of 100%; this effect was observed most clearly for DBTO_2_. The best results for the degree of sulfone conversion and sulfide yield were obtained using AS III.

Biocatalysts consisting of different ASs immobilized in the PVA cryogel (up to 2.5 times) converted sulfones to sulfide more effectively than those used in suspension. The results obtained with AS I were weak, therefore, this kind of AS was not used in subsequent experiments in combination with anaerobic cells of individual cultures.

Further addition of immobilized SRB (*Desulfovibrio vulgaris*) to the medium with immobilized AS II or AS III in the absence of specially introduced H_2_-producing bacteria (*Clostridium acetobutilycum*) did not improve sulfide yield (Table 1 and Table 2). It was established that the conversion of sulfones to sulfide could be intensified with the simultaneous introduction in the medium containing AS III of additional biocatalysts in the form of immobilized SRB (*D. vulgaris*) and H_2_-producing bacteria (*C. acetobutilycum*). Therefore, for 0.15 mM and 0.45 mM DBTO_2_, the maximum sulfide yield after eight days was increased by 10% and 35.5%, respectively. Thus, using a combination of immobilized cells, which consisted by mass of 80% AS III, 10% *D. vulgaris*, and 10% *C. acetobutilycum*, it is possible to completely convert the sulfones to sulfide oin eight days.

### 2.2. Estimation of the Energetic Status of Anaerobic Cells in Sulfons Bioconversion within Methanogenesis

The viability of anaerobic cells in the bioconversion of sulfones was monitored by determining the concentration of intracellular adenosine triphosphate (ATP) (Table 3). When comparing the level of ATP before and after the process, a decrease in the concentration of ATP in the cells was observed in all AS samples. Immobilized cells had a higher residual level of intracellular ATP in comparison with suspended cells. Since the ATP concentration determined for each AS reflects the general state of metabolic processes in the cells, this result indicated that the metabolic activity of the immobilized cells was certainly higher than that of the cells in suspension.

In general, the changes in the concentration of intracellular ATP in anaerobic cells used as biocatalysts in sulfones bioconversion suggest that these cells can be used repeatedly in a similar process. These data were important for further experiments with several working cycles of biocatalysts combinations.

### 2.3. Accumulation of Biogas under the Action of Immobilized AS in the Methanogenesis of Glucose Conducted in the Presence of Sulfones

**Figure 2:**
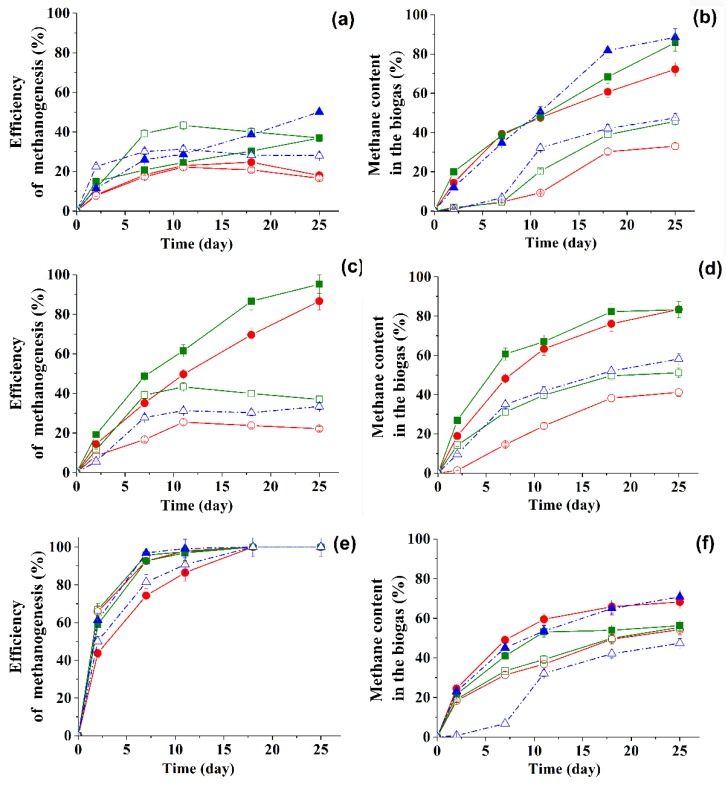
Efficiency of methanogenesis and amount of methane in the accumulated biogas in the course of transformation of 1 g/L glucose and BTO_2_ 0.15 mM (circles) or DBTO_2_ 0.15 mM (squares), 0.45 mM (triangles) under the action of free (open symbols) active sludge or PVA cryogel- immobilized (filled symbols) active sludge of Type I (**a**,**b**), Type II (**c**,**d**), Type III (**e**,**f**).

Efficiency of methanogenesis and amount of methane in the biogas in the course of the transformation of 1g/L of glucose in the presence of BTO_2_ or DBTO_2_ under the action of different ASs, in suspended form and immobilized in the PVA cryogel, were analyzed (Figure 2). For all three types of natural anaerobic consortia, the portion of methane in the composition of biogas produced by immobilized producers was higher than for suspension cells

This is consistent with the previously obtained data for the regular metanogenesis process [14]. The presence of hydrogen in biogas was recorded only during the initial four days, and its content in the biogas composition was no more than 2%. Hydrogen sulfide in the gas phase was not recorded at all.

When evaluating the metabolic activity of natural anaerobic consortia in terms of biogas accumulation in the presence of sulfones, it was noted that AS II and AS III functioned more effectively in this case. So, for AS III, 100% efficiency of methanogenesis in the presence of DBTO_2_ was achieved in just eight days (Figure 2e). Since then, the duration of the repetitive working cycles for further experiments was chosen equal to eight days. Another conclusion from this part of work was that to obtain biogas combined with the bioconversion of sulfones, it is best to use an anaerobic biocatalyst whose microbial composition is close to that of AS III.

### 2.4. Biogas Production under the Action of Combinations of Immobilized Biocatalysts during the Transformation of Glucose and Organic Sulfones under Batch Conditions

**Figure 3:**
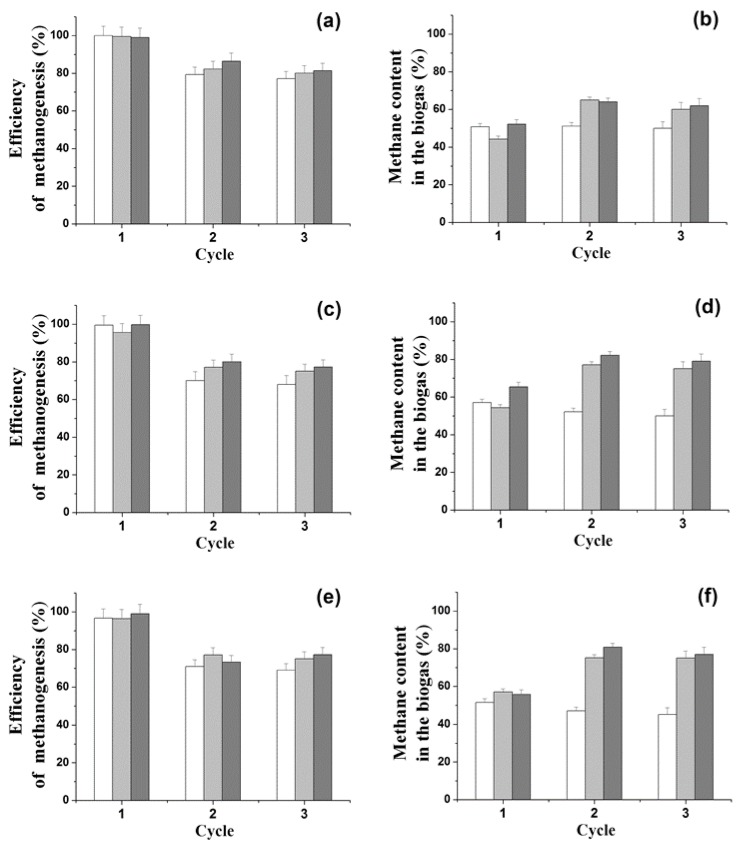
Efficiency of methanogenesis and amount methane in the biogas in the course of transformation of 1 g/L glucose in medium containing 0.15 mM BTO_2_ (**a**,**b**), 0.15 mM DBTO_2_ (**c**,**d**), or 0.45 mM DBTO_2_ (**e**,**f**) under the action of different anaerobic biocatalysts immobilized in the PVA cryogel: 90% AS III + 10% *D. vulgaris* (white columns), 90% AS III + 10% *C. acetobutilycum* (light gray columns), 80% AS III + 10% *D. vulgaris* + 10% *C. acetobutilycum* (dark gray columns). The duration of each single working cycle was eight days. The immobilized biocatalysts were combined in specific mass ratios directly in the reaction medium.

It was shown for the first time that during the conversion of organic sulfones (BTO_2_ or DBTO_2_) to sulfide, it is possible to obtain biogas under batch anaerobic conditions with the joint use of various biocatalysts immobilized in the PVA cryogel: AS (Type III) with SRB (*D. vulgaris*) or/and H_2_-producing bacterium (*C. acetobutilycum*) (Figure 3).

Regardless of the type of sulfone, the addition of SRB or H_2_-producing bacteria to the medium with ASIII did not produce significant changes in the efficiency of methanogenesis and in the amount of methane in the biogas (Figure 2 and Figure 3).

The addition of both SRB and H_2_-producing bacteria made it possible to increase the efficiency of methanogenesis to 95.6%–100% and reduce the duration of the period necessary to reach these values to eight days (Figure 2 and Figure 3).

When only *C. acetobutilycum* cells were added to AS III, the accumulation of hydrogen was two times higher as compared to that obtained with the addition of other variants. However, as a percentage of the composition of biogas, the portion of hydrogen did not exceed 1%.

In the second working cycle, the ratio of methane in the composition of the produced biogas increased if *C. acetobutilycum* cells were added to the AS III, and that phenomenon was probably related to the adaptation of methanogens to the culture conditions, including the presence of sulfones in the medium. This was confirmed by the fact that, in the third cycle of cell usage, the same characteristics of methanogenesis were observed as in the second cycle. The observed effects were associated with the improvement of the adaptation of bacterial immobilized biocatalysts to the culture conditions, as observed in other studies [13,15,16,17].

## 3. Discussion

In the initial planning of the experiments, it was obvious that the characteristics of the sulfone-to-sulfide conversion process would depend on the type of AS samples, with different natural microbial compositions. For this reason, several different ASs, obtained from absolutely different sources, were considered (Table 4).

The sample of AS I was obtained from the wastewater treatment of a potato processing plant [14]. It is known that the initial microbial composition of an AS influences its activity, especially the methanogenic one [18]. A previous genetic analysis of AS I showed the presence in it of cells of the following genera: *Proteobacteria, Bacteroidetes, Chloroflexi, Firmicutes, Verrucomicrobia, Lentisphaerae, Spirochaetales, Planctomycetes, Methanomicrobiales, Methanobacteriales,* and *Methanosarcinales* [19].

The sample of AS II was obtained from a manure treatment digester and had a microbial composition close to the one described previously [14]. Such bacterial consortia are well studied and widely used in methanogenesis processes. The AS formed in the course of methanogenesis in manure (AS II) contained the major bacteria belonging to the phyla *Firmicutes*, *Clostridia,* and *Bacteroidetes*, whereas the archaeal community was dominated by the methanogenic archaea of the taxa *Methanomicrobiales*, *Methanosarcinales*, and *Methanobacteria* [20,21].

The sample of AS III was obtained from methane tanks used for processing alcohol bards containing a large amount of sulfates. This consortium contained representatives of *Clostridiales* and *Desulfobulbus* cells [11] and, as a result, it was characterized by the most efficient conversion of sulfones. Combining natural AS with pure anaerobic cultures is one of the effective methods to influence the characteristics of the methanogenic process. In our case, the target products were biogas and inorganic sulfide. This explains the choice of SRB and H_2_-producing bacteria introduced into the digesters as additional biocatalysts. However, it is also important to evaluate the toxicity of the substrate to selected biocatalysts. The concentration of intracellular ATP is a universal indicator of the viability of living cells. Analysis of this indicator allows to evaluate the inhibitory effect of system components on the metabolic activity of cells in biocatalysts in general. Compared to other biocatalysts, the smallest change in ATP level was observed for AS III and SRB such as *D. vulgaris* cells (Table 3). These data may indirectly indicate that these biocatalysts are the most tolerant to the toxic effects of sulfones. The concentration of intracellular ATP changed most significantly in *C. acetobutilycum* cells. However, this might have been due to insufficient nutrient concentrations for these biocatalysts initially introduced into the culture fluid during experimental studies. Nevertheless, when alone (without AS III), these cells did not “feel comfortable” under the conditions of the examined processes.

Since SRB are known to convert intermediate S-containing compounds to sulfide, the initial use of a pure culture of *D. vulgaris* in this work seemed the best option for the conversion of S-containing organic compounds to sulfide. Indeed, at a concentration of sulfones of 0.15 mM, a pure SRB culture allowed us to achieve maximum values for the degree of sulfone conversion to sulfide (Table 2). However, at a concentration of 0.45 mM DBTO_2_, similar results were not achieved. Thus, the use of a pure culture of SRB in the process under study appeared to be impractical.

It should also be noted that SRB cells have low growth rates, and the accumulation of the biomass necessary for biocatalyst production on a large scale when organizing biotechnological process for sulfone conversion can be time- and energy-consuming. On the basis of the results obtained, it was concluded that it is more appropriate to add SRB cells together with H_2_-producing bacteria to AS III at a concentration of 10% of the total mass of biocatalysts to improve the characteristics of the process under study.

The use of biocatalysts in the immobilized form in the course of the proposed conversion of sulfones to sulfide intensified the processes of methanogenesis. The introduction of additional cultures to the medium with AS III enabled to reduce the time necessary to achieve 100% efficiency of methanogenesis up to eight days, regardless of the added cells. At the same time, AS III cells disposed of DBTO_2_ and BTO_2_ faster with the addition of *D. vulgaris* and *C. acetobutilycum* cells, respectively (Figure 3).

So, for the first time, methanogenic cell associations were used for the simultaneous conversion of S-containing organic sulfones to sulfides under anaerobic conditions and production of biogas. During the implementation of the applied approach to the transformation of sulfones under anaerobic conditions, methanogenesis took place in a medium based on 0.1 M phosphate buffer (pH 7.2). This allowed to solve the problem of the removal of hydrogen sulfide from the resulting biogas. Biogas with a high methane content (60%–80%) was obtained without H_2_S impurities. This fact increases the practical significance of the results obtained in terms of the further use of the produced biogas, which was actually a mixture of CO_2_ and CH_4_ similar to natural gas e (Figure 3).

One of the advantages of using cells immobilized in a PVA cryogel demonstrated in this process is the possibility of the repeated use of these cells without losing efficacy, which was previously demonstrated only for other cells [13,15,16,17]. The results obtained open up prospects for a more in-depth study of the proposed approaches and may be useful to researchers engaged in the study of issues of environmentally safe and cost-effective waste treatment with the production of commercially significant products.

## 4. Materials and Methods

### 4.1. Materials

Poly(vinyl alcohol) type 16/1 (84 kDa) was purchased from Sinopec Corp. (Beijing, China).

To prepare BTO_2_ and DBTO_2_ as starting compounds, we used toluene (ultrapure grade, Reakhim, Moscow, Russia), benzothiophene (98%, Alfa Aesar, Karlsruhe, Germany), dibenzothiophene (98%, Sigma–Aldrich, Saint Louis, MO, USA), hydrogen peroxide (50%, Prime Chemicals Group, Moscow, Russi), and formic acid (88%, chemically pure grade, Komponent-Reaktiv, Moscow, Russia). The starting benzothiophene or dibenzothiophene was added in an amount corresponding to 5000 ppm concentration in a temperature-controlled reactor with *n*-octane.

After dissolution, a 50% hydrogen peroxide solution was added in an amount corresponding to the molar ratio H_2_O_2_/S = 10:1. Formic acid taken in the molar ratio HCOOH/S = 5:1 was used as a catalyst. The mixture was stirred for 2 h at 60 °C. The precipitate was filtered off, washed with excess n-hexane, and dried on a rotary evaporator to constant weight. The obtaining of BTO_2_ and DBTO_2_ was confirmed by GLC and ^1^H-NMR [22,23]. The resulting sulfones were dissolved in ethanol and introduced into the medium for conversion to sulfide within the methanogenesis process.

### 4.2. Microorganisms and Culture Conditions

The AS samples used for immobilization into the PVA cryogel were taken from various sources (Table 4). The dry weight, ash content, and volatile suspended solids (VSS) in the biomass were determined as described previously [24,25].

The bacterial cultures *C. acetobutylicum* B1787 and *D. vulgaris* strain B4053 were obtained from the Russian National Collection of Industrial Microorganisms (www.genetika.ru) and the collection of the Vinogradsky Institute of Microbiology (www.fbras.ru/en/about/institutyi-tsentra/institut-mikrobiologii), respectively, for immobilization in the PVA cryogel and introduction into the anaerobic reactors in addition to the samples of ASs.

To accumulate biomass for the study, the *C. acetobutylicum* strain B1787 was cultivated in the following medium: glucose, 20 g/L; triptone, 10 g/L; yeast extract 5 g/L (pH 6.8). The *D. vulgaris* strain B4053 was cultivated in Postgate medium [26,27].

The bacterial cells were centrifuged for 15 min at 8000 rpm and used for immobilization.

### 4.3. Immobilization of the Cells via Inclusion into the PVA Cryogel

Cells of all bacterial cultures and samples of ASs were immobilized into the PVA cryogel according to previously developed techniques [14,28,29,30]. To realize that, the biomass precipitate was thoroughly mixed with a 10% (*w/v*) aqueous PVA solution to obtain a 10% (*w/w*) concentration of bacterial cells, and a 30% concentration (*w/w*) of anaerobic sludge. This mixture was pipetted into 96-well microplates, which were placed in a freezer at −20 °C for 24 h and then thawed. The granules of PVA cryogel formed in this way contained cells immobilized by inclusion.

### 4.4. Anaerobic Fermentation

Glucose (1 g/L) was used as the main carbon source for methanogenesis, and pH 7.2 was maintained through the use of a 0.1 M K-phosphate buffer. Glucose concentration was determined by an enzymatic technique using a standard Impact reagent kit (“OOO Impact”, Moscow, Russia). Complete consumption of glucose by the biocatalysts occurred in 6 h.

The initial inoculum concentration in batch reactors was 10% (*v/v*) for the suspended forms of AS samples. The quantity of the immobilized AS samples introduced into the medium was such as to ensure similar concentrations of sludge biomass in the liquid phase. The anaerobic incubation was carried out at 35 °C in all experiments.

To study the bioconversion of the sulfones, the solutions were diluted with 0.1 M phosphate buffer (pH 7.2) and loaded into hermetically sealable vials (“anaerobic reactors”, 120 mL). The experiments were performed in triplicate.

A control experiment similar to that described above was concurrently conducted to account for biogas formation due to the possible lysis of the microbial inoculum [14]. Phosphate buffer (0.1 M, pH 7.2) was used instead of the sulfones’ solutions as a control. The methane content in the biogas in the experimental control batches was subtracted from that obtained in the corresponding test batches to calculate the efficiency of methanogenesis.

### 4.5. Accumulation of Biogas and Determination of Its Composition

The total pressure and gases concentration in the gas phase of each reactor were controlled. Gas measurements were repeated until a constant methane content was reached in the gas phase of the reactor. The content of hydrogen, methane, carbon dioxide, and hydrogen sulfide in the gas phase was measured with an LKhM 8 MD chromatograph (Russia) Model 3 with a katharometer (the carrier gas was argon, with 20 mL/min flow rate). Columns, 2 m long, were filled with Q porapak [14]. The oven temperature was maintained at 50 °C, the retention times of hydrogen, methane, carbon dioxide, and hydrogen sulfide were 43, 67, 82, and 271 s, respectively.

### 4.6. Determination of the Concentrations of Sulfide Ions and Sulfones

The concentration of sulfide ions in liquid phase was monitored spectrophotometrically at 660 nm using Shimadzu UV-1202 (Shimadzu, Kyoto, Japan) [31].

The control concentrations of sulfones and the purity of the starting sulfone materials were analyzed by gas chromatography using a “Crystal-2000M” chromatograph with a flame ionization detector, column–Zebron L = 30 m, d = 0.32 mm, liquid phase ZB-1, while programming the temperature from 100 °C to 250 °C. Chromatograms were recorded and analyzed on a computer using the Chromatech Analytic 1.5 program.

### 4.7. Determination of ATP Concentrations in Microbial Cells

The concentration of intracellular ATP in suspended and immobilized cells was determined by the bioluminescent luciferin–luciferase method. For this purpose, granules or biomass were weighed (0.15–0.05 g), transferred to dimethyl sulfoxide (1 mL), and incubated at 25 °C for 1 h to extract intracellular ATP [26]. The luminescence of the samples was registered by a 3560 microluminometer (New Horizons Diagnostics Co., Columbia, MD, USA).

### 4.8. Calculations

The efficiency of methanogenesis (%) and the amount of methane in the biogas (%) were calculated as described previously [14].

The degree of sulfone conversion (%) was calculated as the ratio of the initial concentration of sulfone to the final one in the media and was expressed as a percentage.

Sulfide yield (%) was calculated as the ratio of the initial concentration of sulfide ion to the concentration obtained with the full conversion of the sulfone and expressed as a percentage.

The results shown are the means of at least three independent experiments ± standard deviation (±SD). Statistical analysis was realized using SigmaPlot 12.5 (ver. 12.5, Systat Software Inc., San Jose, CA, USA). Significant differences (*p* ≤ 0.05) between the results were estimated by one-way analysis of variance (ANOVA).

## 5. Conclusions

For the first time, the simultaneous conversion of S-containing organic sulfones into inorganic sulfides under anaerobic conditions and biogas production were obtained. The process was characterized by the accumulation of biogas with a high methane content and without H_2_S impurities. Sulfide accumulated in the medium. It was shown that the immobilized cell biocatalysts used in this work can be reused in the process described. It appeared that, by combining various anaerobic biocatalysts immobilized in a PVA cryogel (AS III with SRB and/or H_2_-producing bacteria), it is possible to achieve 99.7%–100% efficiency of methanogenesis and 97.8%–100% conversion of BTO_2_ and DBTO_2_ (up to 0.45 mM) to sulfide in eight days. Probably, in the future, by optimizing the conditions of such process, it will be possible to even increase the concentrations of organic sulfones that can be used in the conversion.

In general, the results obtained open up prospects for a more in-depth study of the proposed processes and may be useful to researchers studying the improvement of the environmental safety of processes to obtain commercially significant products, in particular, lacking organic S-containing compounds.

After further research, the proposed solution may have practical significance for the conversion of organic sulfones into sulfide in large extracts after oxidative desulfurization of fuel oils [22,32]. Oxidative desulfurization is now offered as a promising approach for implementing the deep desulfurization of oil fractions [8]. At the same time, ways for the further transformation of organic S-containing compounds after their extraction from oil remain to be discovered.

## Figures and Tables

**Table 1:** Parameters of sulfones bioconversion to sulfide under the action of different samples of anaerobic sludge (AS) (S and Im: suspended and immobilized forms of AS, respectively).

Biocatalyst	Form	Degree of Sulfone Conversion, %		Sulfide Yield, % ^1^	
		The 8th Day	The 18th Day	The 8th Day	The 18th Day
		**0.15 mM BTO_2_**			
AS I	S	100	100	40.3 ± 1.9	87.9 ± 2.3
	Im	100	100	97.8 ± 2.9	100
AS II	S	100	100	68.1 ± 2.4	99.1 ± 0.9
	Im	100	100	98.4 ± 1.6	100
AS III	S	100	100	100	100
	Im	100	100	100	100
		**0.15 mM DBTO_2_**			
AS I	S	100	100	26.4 ± 1.3	56.8 ± 1.8
	Im	100	100	27.1 ± 1.3	61.4 ± 2.1
AS II	S	100	100	70.2 ± 2.5	99.1 ± 0.9
	Im	100	100	70.7 ± 2.5	100
AS III	S	100	100	63.4 ± 2.1	98.4 ± 0.9
	Im	100	100	87.9 ± 2.3	100
		**0.45 mM DBTO_2_**			
AS I	S	99.4 ± 0.2	100	26.6 ± 1.3	64.2 ± 2.2
	Im	98.1 ± 0.2	100	26.7 ± 1.3	73.1 ± 2.6
AS II	S	100	100	39.9 ± 1.9	67.8 ± 2.4
	Im	98.1 ± 0.2	100	57.6 ± 2.5	97.5 ± 2.8
AS III	S	99.1 ± 0.2	100	39.9 ± 1.9	100
	Im	99.1 ± 0.2	100	64.2 ± 2.2	100

^1^ 100% Sulfide yield corresponds to inorganic sulfide concentration of 4.7 ± 0.1 mg/L and 13.7 ± 0.7 mg/L for 0.15 mM and 0.45 mM concentration of sulfone, respectively.

**Table 2:** Parameters of organic sulfones bioconversion to sulfide under the action of different biocatalysts immobilized in the PVA cryogel.

Biocatalysts and Their Combinations	Degree of Sulfone Conversion, %		Sulfide Yield, % ^1^	
	The 8th Day	The 18th Day	The 8th Day	The 18th Day
	**0.15 mM BTO_2_**			
*Desulfovibri vulgaris*	100	100	100	100
90% AS II + 10% *D. vulgaris*	100	100	98.4 ± 1.4	100
90% AS III + 10% *D. vulgaris*	100	100	100	100
90% AS III + 10% *Clostridium acetobutilycum*	100	100	100	100
80% AS III + 10% *D. vulgaris* + 10% *C. acetobutilycum*	100	100	100	100
	**0.15 mM DBTO_2_**			
*D. vulgaris*	100	100	97.8 ± 1.7	100
90% AS II + 10% *D. vulgaris*	100	100	62.1 ± 2.4	100
90% AS III + 10% *D. vulgaris*	100	100	83.2 ± 3.1	100
90% AS III + 10% *C. acetobutilycum*	100	100	88.5 ± 3.4	100
80% AS III + 10% *D. vulgaris* + 10% *C. acetobutilycum*	100	100	91.8 ± 4.1	100
	**0.45 mM DBTO_2_**			
*D. vulgaris*	99.4 ± 0.2	100	73.1 ± 2.6	100
90% AS II + 10% *D. vulgaris*	99.1 ± 0.2	100	48.7 ± 2.3	97.5 ± 2.1
90% AS III + 10% *D. vulgaris*	99.1 ± 0.2	100	84.2 ± 3.2	99.7 ± 0.3
90% AS III + 10% *C. acetobutilycum*	100	100	100	100
80% AS III + 10% *D. vulgaris* + 10% *C. acetobutilycum*	100	100	99.7 ± 0.3	100

^1^ 100% Sulfide yield corresponds to inorganic sulfide concentration of 4.7 ± 0.1 mg/L and 13.7 ± 0.7 mg/L for 0.15 mM and 0.45 mM concentration of sulfone, respectively.

**Table 3:** Concentration of intracellular ATP in anaerobic cells before (control) and after their usage in sulfones bioconversion for 18 days.

Biocatalyst	Form	Concentration of Intracellular ATP, × 10^−12^ mol/mg DCW ^1^			
		Control	0.15 mM BTO_2_	0.15 mM DBTO_2_	0.45 mM DBTO_2_
AS I	S	50.2 ± 1.8	1.5 ± 0.1	3.2 ± 0.2	2.9 ± 0.1
	Im	55.2 ± 1.8	5.7 ± 0.2	25.1 ± 0.9	4.1 ± 0.2
AS II	S	44.6 ± 1.4	4.3 ± 0.2	4.2 ± 0.2	9.1 ± 0.4
	Im	36.5 ± 1.4	16.9 ± 0.7	19.4 ± 0.7	n/d^2^
AS III	S	55.7 ± 1.9	17.1 ± 0.7	25.7 ± 0.9	33.5 ±1.3
	Im	52.3 ± 1.9	27.3 ± 1.1	20.8 ± 0.8	31.1 ± 1.2
*D. vulgaris*	Im	7.1 ± 0.2	4.1 ± 0.2	4.1 ± 0.2	n/d
*C. acetobutilycum*	Im	66.1 ± 3.1	12.1 ± 0.5	11.1 ± 0.4	18.2 ± 0.7

^1^ DCW: dry cell weight, ^2^ n/d: no data.

**Table 4:** Characteristics of the different AS samples used in this study. VSS: volatile suspended solids.

AS-Sample	Source ^1^	Dry Matter, g/L	Ash, %	Biomass VSS, g/L
I	Frito Lay plant (Kashira,)	62.9 ± 3.1	44.3 ± 0.8	34.7 ± 1.2
II	Methane tank processing cattle farm waste (Dmitrov)	44.6 ± 1.5	35.8 ± 1.1	28.6 ± 0.1
III	Brincalov plant (Dolgoprudniy)	79.9 ± 3.6	37.9 ± 1.7	49.6 ± 1.4

^1^ All sources of methanogenic sludge are located in Moscow region, Russia.
